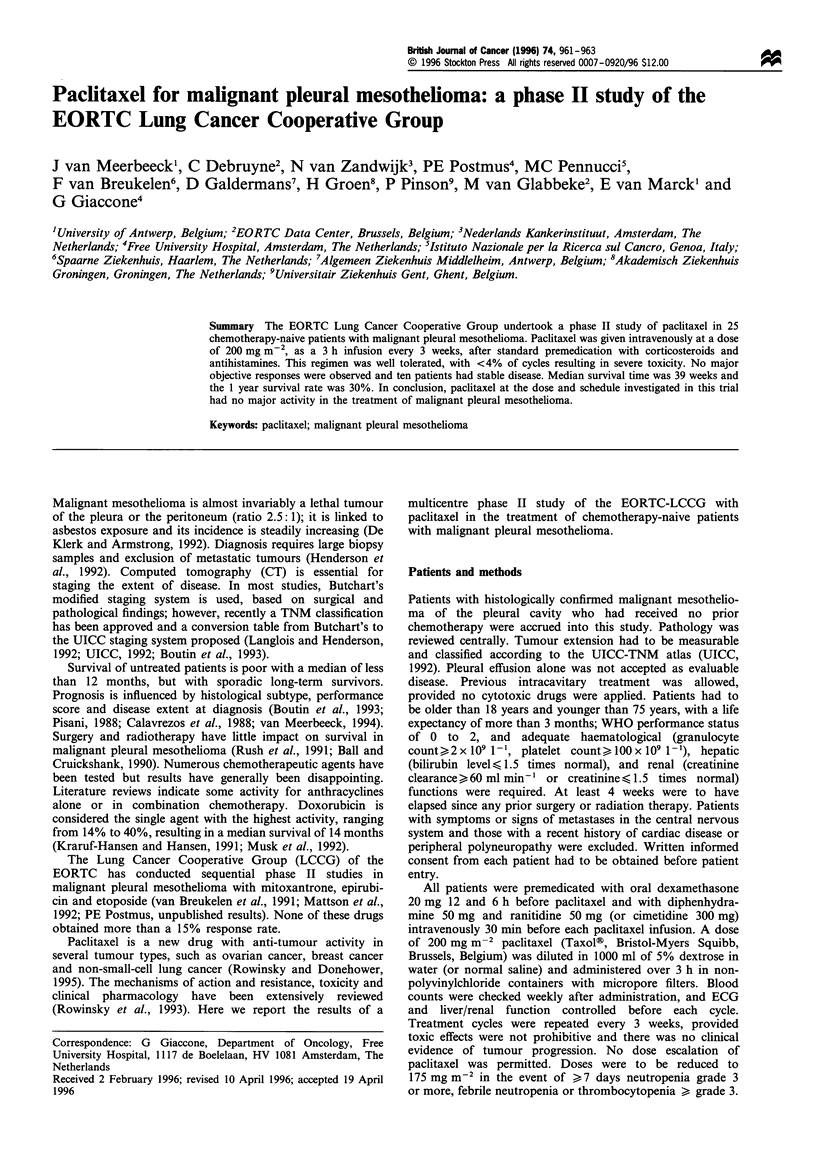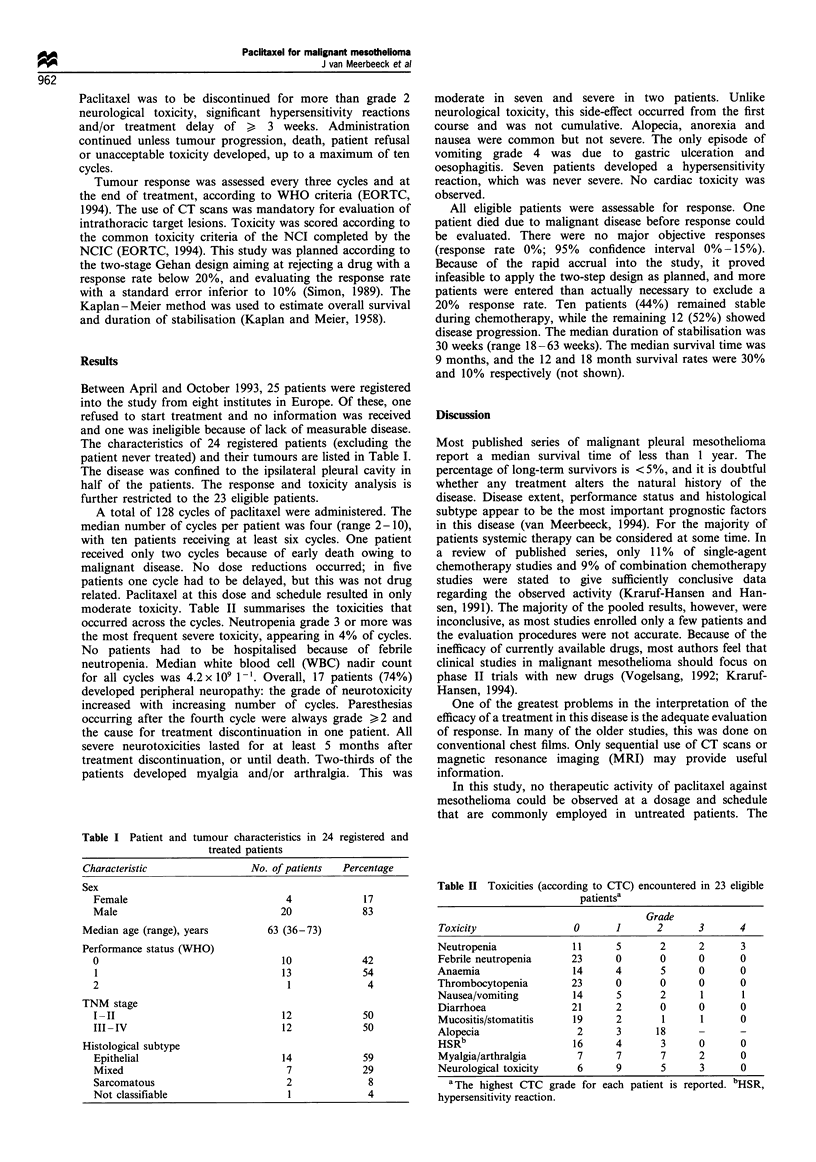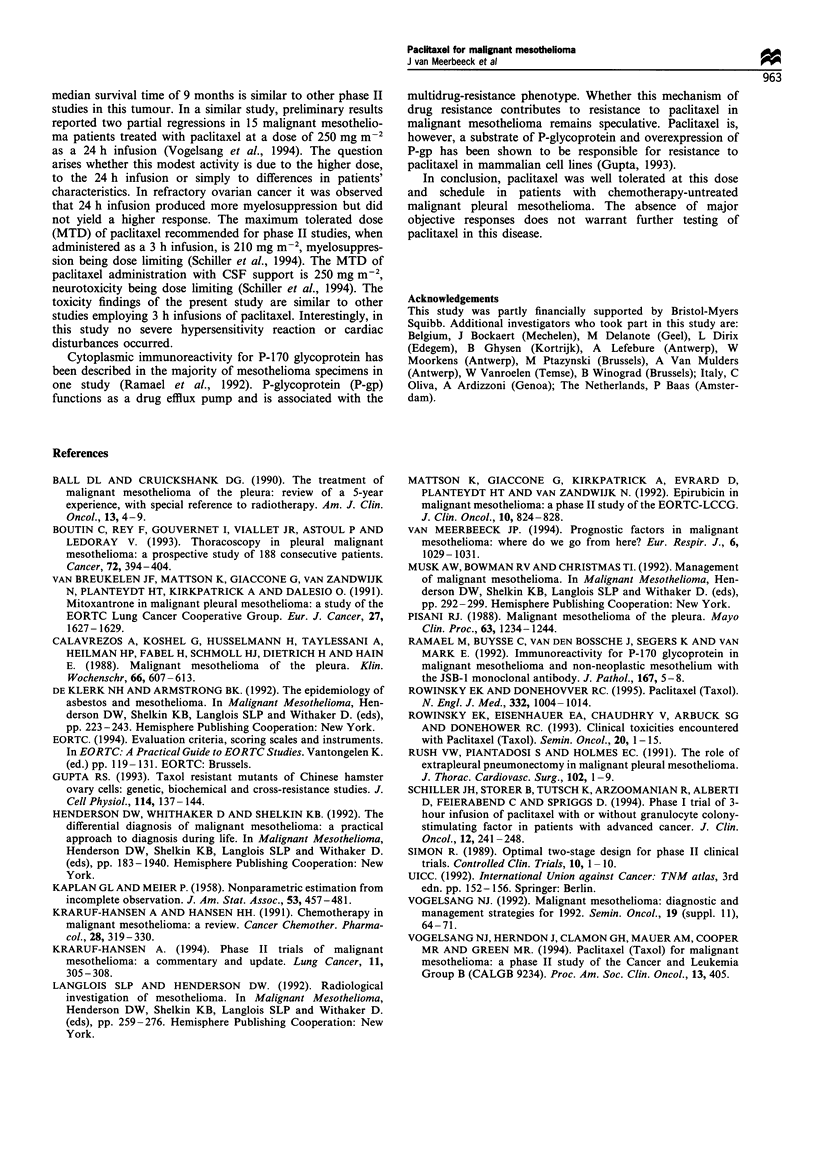# Paclitaxel for malignant pleural mesothelioma: a phase II study of the EORTC Lung Cancer Cooperative Group.

**DOI:** 10.1038/bjc.1996.465

**Published:** 1996-09

**Authors:** J. van Meerbeeck, C. Debruyne, N. van Zandwijk, P. E. Postmus, M. C. Pennucci, F. van Breukelen, D. Galdermans, H. Groen, P. Pinson, M. van Glabbeke, E. van Marck, G. Giaccone

**Affiliations:** University of Antwerp, Belgium.

## Abstract

The EORTC Lung Cancer Cooperative Group undertook a phase II study of paclitaxel in 25 chemotherapy-naive patients with malignant pleural mesothelioma. Paclitaxel was given intravenously at a dose of 200 mg m-2 as a 3 h infusion every 3 weeks, after standard premedication with corticosteroids and antihistamines. This regimen was well tolerated, with < 4% of cycles resulting in severe toxicity. No major objective responses were observed and ten patients had stable disease. Median survival time was 39 weeks and the 1 year survival rate was 30%. In conclusion, paclitaxel at the dose and schedule investigated in this trial had no major activity in the treatment of malignant pleural mesothelioma.


British Journal of Cancer (1996) 74, 961-963
© 1996 Stockton Press All rights reserved 0007-0920/96 $12.00

### Paclitaxel for malignant pleural mesothelioma: a phase II study of the EORTC Lung Cancer Cooperative Group

J van Meerbeeck[1], C Debruyne[2], N van Zandwijk[3], PE Postmus[4], MC Pennucci[5], F van Breukelen[6], D Galdermans[7], H Groen[8], P Pinson[9], M van Glabbeke[2], E van Marck[1] and G Giaccone[4]

[1]*University of Antwerp, Belgium;* [2]*EORTC Data Center, Brussels, Belgium;* [3]*Nederlands Kankerinstituut, Amsterdam, The Netherlands;* [4]*Free University Hospital, Amsterdam, The Netherlands;* [5]*Istituto Nazionale per la Ricerca sul Cancro, Genoa, Italy;* [6]*Spaarne Ziekenhuis, Haarlem, The Netherlands;* [7]*Algemeen Ziekenhuis Middlelheim, Antwerp, Belgium;* [8]*Akademisch Ziekenhuis Groningen, Groningen, The Netherlands;* [9]*Universitair Ziekenhuis Gent, Ghent, Belgium.*

**Summary** The EORTC Lung Cancer Cooperative Group undertook a phase II study of paclitaxel in 25 chemotherapy-naive patients with malignant pleural mesothelioma. Paclitaxel was given intravenously at a dose of 200 mg $m^{-2}$, as a 3 h infusion every 3 weeks, after standard premedication with corticosteroids and antihistamines. This regimen was well tolerated, with <4% of cycles resulting in severe toxicity. No major objective responses were observed and ten patients had stable disease. Median survival time was 39 weeks and the 1 year survival rate was 30%. In conclusion, paclitaxel at the dose and schedule investigated in this trial had no major activity in the treatment of malignant pleural mesothelioma.

**Keywords:** paclitaxel; malignant pleural mesothelioma

Malignant mesothelioma is almost invariably a lethal tumour of the pleura or the peritoneum (ratio 2.5:1); it is linked to asbestos exposure and its incidence is steadily increasing (De Klerk and Armstrong, 1992). Diagnosis requires large biopsy samples and exclusion of metastatic tumours (Henderson *et al.*, 1992). Computed tomography (CT) is essential for staging the extent of disease. In most studies, Butchart's modified staging system is used, based on surgical and pathological findings; however, recently a TNM classification has been approved and a conversion table from Butchart's to the UICC staging system proposed (Langlois and Henderson, 1992; UICC, 1992; Boutin *et al.*, 1993).

Survival of untreated patients is poor with a median of less than 12 months, but with sporadic long-term survivors. Prognosis is influenced by histological subtype, performance score and disease extent at diagnosis (Boutin *et al.*, 1993; Pisani, 1988; Calavrezos *et al.*, 1988; van Meerbeeck, 1994). Surgery and radiotherapy have little impact on survival in malignant pleural mesothelioma (Rush *et al.*, 1991; Ball and Cruickshank, 1990). Numerous chemotherapeutic agents have been tested but results have generally been disappointing. Literature reviews indicate some activity for anthracyclines alone or in combination chemotherapy. Doxorubicin is considered the single agent with the highest activity, ranging from 14% to 40%, resulting in a median survival of 14 months (Kraruf-Hansen and Hansen, 1991; Musk *et al.*, 1992).

The Lung Cancer Cooperative Group (LCCG) of the EORTC has conducted sequential phase II studies in malignant pleural mesothelioma with mitoxantrone, epirubicin and etoposide (van Breukelen *et al.*, 1991; Mattson *et al.*, 1992; PE Postmus, unpublished results). None of these drugs obtained more than a 15% response rate.

Paclitaxel is a new drug with anti-tumour activity in several tumour types, such as ovarian cancer, breast cancer and non-small-cell lung cancer (Rowinsky and Donehower, 1995). The mechanisms of action and resistance, toxicity and clinical pharmacology have been extensively reviewed (Rowinsky *et al.*, 1993). Here we report the results of a multicentre phase II study of the EORTC-LCCG with paclitaxel in the treatment of chemotherapy-naive patients with malignant pleural mesothelioma.

Correspondence: G Giaccone, Department of Oncology, Free University Hospital, 1117 de Boelelaan, HV 1081 Amsterdam, The Netherlands

Received 2 February 1996; revised 10 April 1996; accepted 19 April 1996

### Patients and methods

Patients with histologically confirmed malignant mesothelioma of the pleural cavity who had received no prior chemotherapy were accrued into this study. Pathology was reviewed centrally. Tumour extension had to be measurable and classified according to the UICC-TNM atlas (UICC, 1992). Pleural effusion alone was not accepted as evaluable disease. Previous intracavitary treatment was allowed, provided no cytotoxic drugs were applied. Patients had to be older than 18 years and younger than 75 years, with a life expectancy of more than 3 months; WHO performance status of 0 to 2, and adequate haematological (granulocyte count $\geq 2 \times 10^9$ $l^{-1}$, platelet count $\geq 100 \times 10^9$ $l^{-1}$), hepatic (bilirubin level $\leq 1.5$ times normal), and renal (creatinine clearance $\geq 60$ ml $min^{-1}$ or creatinine $\leq 1.5$ times normal) functions were required. At least 4 weeks were to have elapsed since any prior surgery or radiation therapy. Patients with symptoms or signs of metastases in the central nervous system and those with a recent history of cardiac disease or peripheral polyneuropathy were excluded. Written informed consent from each patient had to be obtained before patient entry.

All patients were premedicated with oral dexamethasone 20 mg 12 and 6 h before paclitaxel and with diphenhydramine 50 mg and ranitidine 50 mg (or cimetidine 300 mg) intravenously 30 min before each paclitaxel infusion. A dose of 200 mg $m^{-2}$ paclitaxel (Taxol®, Bristol-Myers Squibb, Brussels, Belgium) was diluted in 1000 ml of 5% dextrose in water (or normal saline) and administered over 3 h in non-polyvinylchloride containers with micropore filters. Blood counts were checked weekly after administration, and ECG and liver/renal function controlled before each cycle. Treatment cycles were repeated every 3 weeks, provided toxic effects were not prohibitive and there was no clinical evidence of tumour progression. No dose escalation of paclitaxel was permitted. Doses were to be reduced to 175 mg $m^{-2}$ in the event of $\geq 7$ days neutropenia grade 3 or more, febrile neutropenia or thrombocytopenia $\geq$ grade 3.

Paclitaxel was to be discontinued for more than grade 2 neurological toxicity, significant hypersensitivity reactions and/or treatment delay of ⩾ 3 weeks. Administration continued unless tumour progression, death, patient refusal or unacceptable toxicity developed, up to a maximum of ten cycles.

Tumour response was assessed every three cycles and at the end of treatment, according to WHO criteria (EORTC, 1994). The use of CT scans was mandatory for evaluation of intrathoracic target lesions. Toxicity was scored according to the common toxicity criteria of the NCI completed by the NCIC (EORTC, 1994). This study was planned according to the two-stage Gehan design aiming at rejecting a drug with a response rate below 20%, and evaluating the response rate with a standard error inferior to 10% (Simon, 1989). The Kaplan–Meier method was used to estimate overall survival and duration of stabilisation (Kaplan and Meier, 1958).

### Results

Between April and October 1993, 25 patients were registered into the study from eight institutes in Europe. Of these, one refused to start treatment and no information was received and one was ineligible because of lack of measurable disease. The characteristics of 24 registered patients (excluding the patient never treated) and their tumours are listed in Table I. The disease was confined to the ipsilateral pleural cavity in half of the patients. The response and toxicity analysis is further restricted to the 23 eligible patients.

A total of 128 cycles of paclitaxel were administered. The median number of cycles per patient was four (range 2–10), with ten patients receiving at least six cycles. One patient received only two cycles because of early death owing to malignant disease. No dose reductions occurred; in five patients one cycle had to be delayed, but this was not drug related. Paclitaxel at this dose and schedule resulted in only moderate toxicity. Table II summarises the toxicities that occurred across the cycles. Neutropenia grade 3 or more was the most frequent severe toxicity, appearing in 4% of cycles. No patients had to be hospitalised because of febrile neutropenia. Median white blood cell (WBC) nadir count for all cycles was $4.2 \times 10^9\ l^{-1}$. Overall, 17 patients (74%) developed peripheral neuropathy: the grade of neurotoxicity increased with increasing number of cycles. Paresthesias occurring after the fourth cycle were always grade ⩾2 and the cause for treatment discontinuation in one patient. All severe neurotoxicities lasted for at least 5 months after treatment discontinuation, or until death. Two-thirds of the patients developed myalgia and/or arthralgia. This was moderate in seven and severe in two patients. Unlike neurological toxicity, this side-effect occurred from the first course and was not cumulative. Alopecia, anorexia and nausea were common but not severe. The only episode of vomiting grade 4 was due to gastric ulceration and oesophagitis. Seven patients developed a hypersensitivity reaction, which was never severe. No cardiac toxicity was observed.

All eligible patients were assessable for response. One patient died due to malignant disease before response could be evaluated. There were no major objective responses (response rate 0%; 95% confidence interval 0%–15%). Because of the rapid accrual into the study, it proved infeasible to apply the two-step design as planned, and more patients were entered than actually necessary to exclude a 20% response rate. Ten patients (44%) remained stable during chemotherapy, while the remaining 12 (52%) showed disease progression. The median duration of stabilisation was 30 weeks (range 18–63 weeks). The median survival time was 9 months, and the 12 and 18 month survival rates were 30% and 10% respectively (not shown).

### Discussion

Most published series of malignant pleural mesothelioma report a median survival time of less than 1 year. The percentage of long-term survivors is <5%, and it is doubtful whether any treatment alters the natural history of the disease. Disease extent, performance status and histological subtype appear to be the most important prognostic factors in this disease (van Meerbeeck, 1994). For the majority of patients systemic therapy can be considered at some time. In a review of published series, only 11% of single-agent chemotherapy studies and 9% of combination chemotherapy studies were stated to give sufficiently conclusive data regarding the observed activity (Kraruf-Hansen and Hansen, 1991). The majority of the pooled results, however, were inconclusive, as most studies enrolled only a few patients and the evaluation procedures were not accurate. Because of the inefficacy of currently available drugs, most authors feel that clinical studies in malignant mesothelioma should focus on phase II trials with new drugs (Vogelsang, 1992; Kraruf-Hansen, 1994).

One of the greatest problems in the interpretation of the efficacy of a treatment in this disease is the adequate evaluation of response. In many of the older studies, this was done on conventional chest films. Only sequential use of CT scans or magnetic resonance imaging (MRI) may provide useful information.

In this study, no therapeutic activity of paclitaxel against mesothelioma could be observed at a dosage and schedule that are commonly employed in untreated patients. The

**Table I** Patient and tumour characteristics in 24 registered and treated patients

| *Characteristic* | *No. of patients* | *Percentage* |
|---|---|---|
| Sex | | |
| Female | 4 | 17 |
| Male | 20 | 83 |
| Median age (range), years | 63 (36–73) | |
| Performance status (WHO) | | |
| 0 | 10 | 42 |
| 1 | 13 | 54 |
| 2 | 1 | 4 |
| TNM stage | | |
| I–II | 12 | 50 |
| III–IV | 12 | 50 |
| Histological subtype | | |
| Epithelial | 14 | 59 |
| Mixed | 7 | 29 |
| Sarcomatous | 2 | 8 |
| Not classifiable | 1 | 4 |

**Table II** Toxicities (according to CTC) encountered in 23 eligible patients[a]

| | | | *Grade* | | |
|---|---|---|---|---|---|
| *Toxicity* | *0* | *1* | *2* | *3* | *4* |
| Neutropenia | 11 | 5 | 2 | 2 | 3 |
| Febrile neutropenia | 23 | 0 | 0 | 0 | 0 |
| Anaemia | 14 | 4 | 5 | 0 | 0 |
| Thrombocytopenia | 23 | 0 | 0 | 0 | 0 |
| Nausea/vomiting | 14 | 5 | 2 | 1 | 1 |
| Diarrhoea | 21 | 2 | 0 | 0 | 0 |
| Mucositis/stomatitis | 19 | 2 | 1 | 1 | 0 |
| Alopecia | 2 | 3 | 18 | – | – |
| HSR[b] | 16 | 4 | 3 | 0 | 0 |
| Myalgia/arthralgia | 7 | 7 | 7 | 2 | 0 |
| Neurological toxicity | 6 | 9 | 5 | 3 | 0 |

[a]The highest CTC grade for each patient is reported. [b]HSR, hypersensitivity reaction.

median survival time of 9 months is similar to other phase II studies in this tumour. In a similar study, preliminary results reported two partial regressions in 15 malignant mesothelioma patients treated with paclitaxel at a dose of 250 mg $m^{-2}$ as a 24 h infusion (Vogelsang *et al.*, 1994). The question arises whether this modest activity is due to the higher dose, to the 24 h infusion or simply to differences in patients' characteristics. In refractory ovarian cancer it was observed that 24 h infusion produced more myelosuppression but did not yield a higher response. The maximum tolerated dose (MTD) of paclitaxel recommended for phase II studies, when administered as a 3 h infusion, is 210 mg $m^{-2}$, myelosuppression being dose limiting (Schiller *et al.*, 1994). The MTD of paclitaxel administration with CSF support is 250 mg $m^{-2}$, neurotoxicity being dose limiting (Schiller *et al.*, 1994). The toxicity findings of the present study are similar to other studies employing 3 h infusions of paclitaxel. Interestingly, in this study no severe hypersensitivity reaction or cardiac disturbances occurred.

Cytoplasmic immunoreactivity for P-170 glycoprotein has been described in the majority of mesothelioma specimens in one study (Ramael *et al.*, 1992). P-glycoprotein (P-gp) functions as a drug efflux pump and is associated with the multidrug-resistance phenotype. Whether this mechanism of drug resistance contributes to resistance to paclitaxel in malignant mesothelioma remains speculative. Paclitaxel is, however, a substrate of P-glycoprotein and overexpression of P-gp has been shown to be responsible for resistance to paclitaxel in mammalian cell lines (Gupta, 1993).

In conclusion, paclitaxel was well tolerated at this dose and schedule in patients with chemotherapy-untreated malignant pleural mesothelioma. The absence of major objective responses does not warrant further testing of paclitaxel in this disease.

**Acknowledgements**
This study was partly financially supported by Bristol-Myers Squibb. Additional investigators who took part in this study are: Belgium, J Bockaert (Mechelen), M Delanote (Geel), L Dirix (Edegem), B Ghysen (Kortrijk), A Lefebure (Antwerp), W Moorkens (Antwerp), M Ptazynski (Brussels), A Van Mulders (Antwerp), W Vanroelen (Temse), B Winograd (Brussels); Italy, C Oliva, A Ardizzoni (Genoa); The Netherlands, P Baas (Amsterdam).

### References

BALL DL AND CRUICKSHANK DG. (1990). The treatment of malignant mesothelioma of the pleura: review of a 5-year experience, with special reference to radiotherapy. *Am. J. Clin. Oncol.*, **13**, 4–9.

BOUTIN C, REY F, GOUVERNET I, VIALLET JR, ASTOUL P AND LEDORAY V. (1993). Thoracoscopy in pleural malignant mesothelioma: a prospective study of 188 consecutive patients. *Cancer*, **72**, 394–404.

VAN BREUKELEN JF, MATTSON K, GIACCONE G, VAN ZANDWIJK N, PLANTEYDT HT, KIRKPATRICK A AND DALESIO O. (1991). Mitoxantrone in malignant pleural mesothelioma: a study of the EORTC Lung Cancer Cooperative Group. *Eur. J. Cancer*, **27**, 1627–1629.

CALAVREZOS A, KOSHEL G, HUSSELMANN H, TAYLESSANI A, HEILMAN HP, FABEL H, SCHMOLL HJ, DIETRICH H AND HAIN E. (1988). Malignant mesothelioma of the pleura. *Klin. Wochenschr*, **66**, 607–613.

DE KLERK NH AND ARMSTRONG BK. (1992). The epidemiology of asbestos and mesothelioma. In *Malignant Mesothelioma*, Henderson DW, Shelkin KB, Langlois SLP and Withaker D. (eds), pp. 223–243. Hemisphere Publishing Cooperation: New York.

EORTC. (1994). Evaluation criteria, scoring scales and instruments. In *EORTC: A Practical Guide to EORTC Studies*. Vantongelen K. (ed.) pp. 119–131. EORTC: Brussels.

GUPTA RS. (1993). Taxol resistant mutants of Chinese hamster ovary cells: genetic, biochemical and cross-resistance studies. *J. Cell Physiol.*, **114**, 137–144.

HENDERSON DW, WHITHAKER D AND SHELKIN KB. (1992). The differential diagnosis of malignant mesothelioma: a practical approach to diagnosis during life. In *Malignant Mesothelioma*, Henderson DW, Shelkin KB, Langlois SLP and Withaker D. (eds), pp. 183–1940. Hemisphere Publishing Cooperation: New York.

KAPLAN GL AND MEIER P. (1958). Nonparametric estimation from incomplete observation. *J. Am. Stat. Assoc.*, **53**, 457–481.

KRARUF-HANSEN A AND HANSEN HH. (1991). Chemotherapy in malignant mesothelioma: a review. *Cancer Chemother. Pharmacol.*, **28**, 319–330.

KRARUF-HANSEN A. (1994). Phase II trials of malignant mesothelioma: a commentary and update. *Lung Cancer*, **11**, 305–308.

LANGLOIS SLP AND HENDERSON DW. (1992). Radiological investigation of mesothelioma. In *Malignant Mesothelioma*, Henderson DW, Shelkin KB, Langlois SLP and Withaker D. (eds), pp. 259–276. Hemisphere Publishing Cooperation: New York.

MATTSON K, GIACCONE G, KIRKPATRICK A, EVRARD D, PLANTEYDT HT AND VAN ZANDWIJK N. (1992). Epirubicin in malignant mesothelioma: a phase II study of the EORTC-LCCG. *J. Clin. Oncol.*, **10**, 824–828.

VAN MEERBEECK JP. (1994). Prognostic factors in malignant mesothelioma: where do we go from here? *Eur. Respir. J.*, **6**, 1029–1031.

MUSK AW, BOWMAN RV AND CHRISTMAS TI. (1992). Management of malignant mesothelioma. In *Malignant Mesothelioma*, Henderson DW, Shelkin KB, Langlois SLP and Withaker D. (eds), pp. 292–299. Hemisphere Publishing Cooperation: New York.

PISANI RJ. (1988). Malignant mesothelioma of the pleura. *Mayo Clin. Proc.*, **63**, 1234–1244.

RAMAEL M, BUYSSE C, VAN DEN BOSSCHE J, SEGERS K AND VAN MARK E. (1992). Immunoreactivity for P-170 glycoprotein in malignant mesothelioma and non-neoplastic mesothelium with the JSB-1 monoclonal antibody. *J. Pathol.*, **167**, 5–8.

ROWINSKY EK AND DONEHOVVER RC. (1995). Paclitaxel (Taxol). *N. Engl. J. Med.*, **332**, 1004–1014.

ROWINSKY EK, EISENHAUER EA, CHAUDHRY V, ARBUCK SG AND DONEHOWER RC. (1993). Clinical toxicities encountered with Paclitaxel (Taxol). *Semin. Oncol.*, **20**, 1–15.

RUSH VW, PIANTADOSI S AND HOLMES EC. (1991). The role of extrapleural pneumonectomy in malignant pleural mesothelioma. *J. Thorac. Cardiovasc. Surg.*, **102**, 1–9.

SCHILLER JH, STORER B, TUTSCH K, ARZOOMANIAN R, ALBERTI D, FEIERABEND C AND SPRIGGS D. (1994). Phase I trial of 3-hour infusion of paclitaxel with or without granulocyte colony-stimulating factor in patients with advanced cancer. *J. Clin. Oncol.*, **12**, 241–248.

SIMON R. (1989). Optimal two-stage design for phase II clinical trials. *Controlled Clin. Trials*, **10**, 1–10.

UICC. (1992). *International Union against Cancer: TNM atlas*, 3rd edn. pp. 152–156. Springer: Berlin.

VOGELSANG NJ. (1992). Malignant mesothelioma: diagnostic and management strategies for 1992. *Semin. Oncol.*, **19** (suppl. 11), 64–71.

VOGELSANG NJ, HERNDON J, CLAMON GH, MAUER AM, COOPER MR AND GREEN MR. (1994). Paclitaxel (Taxol) for malignant mesothelioma: a phase II study of the Cancer and Leukemia Group B (CALGB 9234). *Proc. Am. Soc. Clin. Oncol.*, **13**, 405.